# Supplementary material for: Humidified and standard oxygen therapy in acute severe asthma in children (HUMOX): A pilot randomised controlled trial
Source: PLoS One. 2022 Feb 3;17(2):e0263044. doi: 10.1371/journal.pone.0263044 (PMC8812987; doi:10.1371/journal.pone.0263044)
Supplement: S1 Fig — (DOCX) [file pone.0263044.s002.docx]

Supplementary Figure S1: Number of ASS assessments missing/not assessed/assessed at different time points

*Note that for most ASS assessments that are missing, the date and time is also missing and therefore cannot be classified into one of the below categories.*

**
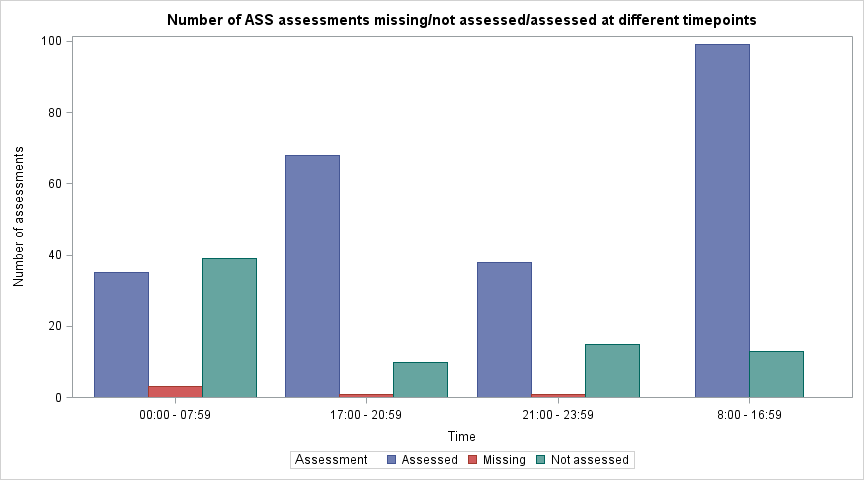
**
