# Supplementary material for: Humidified and standard oxygen therapy in acute severe asthma in children (HUMOX): A pilot randomised controlled trial
Source: PLoS One. 2022 Feb 3;17(2):e0263044. doi: 10.1371/journal.pone.0263044 (PMC8812987; doi:10.1371/journal.pone.0263044)
Supplement: S2 Fig — (DOCX) [file pone.0263044.s003.docx]

Supplementary Figure S2: Number of PRAM assessments missing/not assessed/assessed at different time points

**
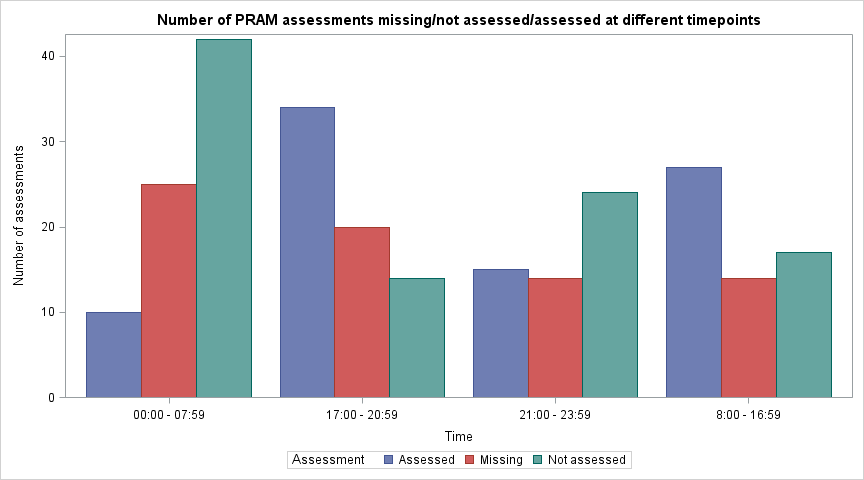
**
