# Supplementary figures and images for: Humidified and standard oxygen therapy in acute severe asthma in children (HUMOX): A pilot randomised controlled trial
Source: PLoS One. 2022 Feb 3;17(2):e0263044. doi: 10.1371/journal.pone.0263044 (PMC8812987; doi:10.1371/journal.pone.0263044)

Supplementary Figure 3: Change in oxygen saturation by treatment group in the first 24 hours


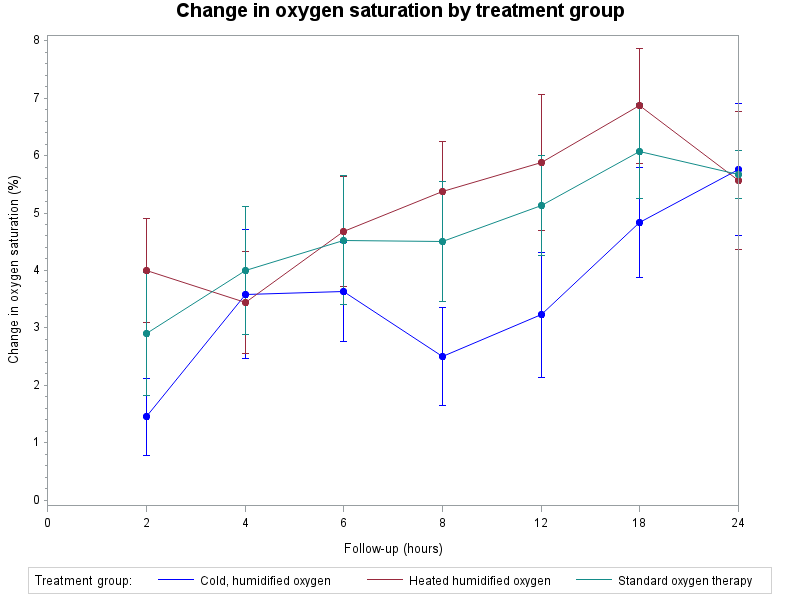

Supplement: S3 Fig — (DOCX) [file pone.0263044.s004.docx]

Supplementary Figure S4: Parental assessment of outcomes


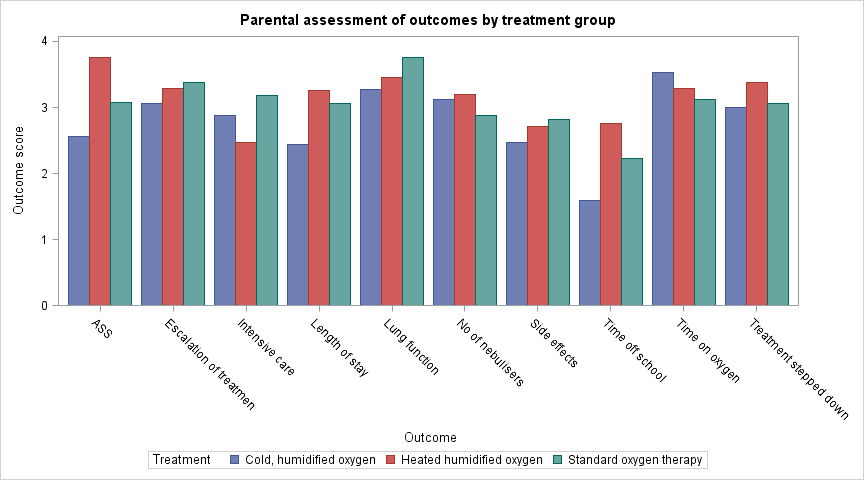

Supplement: S4 Fig — (DOCX) [file pone.0263044.s005.docx]
