# Supplementary material for: Humidified and standard oxygen therapy in acute severe asthma in children (HUMOX): A pilot randomised controlled trial
Source: PLoS One. 2022 Feb 3;17(2):e0263044. doi: 10.1371/journal.pone.0263044 (PMC8812987; doi:10.1371/journal.pone.0263044)
Supplement: S1 Table — (DOCX) [file pone.0263044.s006.docx]

Supplementary Table S1: Reasons for discontinuation of allocated intervention

| Participant status | Date of randomisation | Time of randomisation (24 hour) | Randomised Therapy | Length of time on randomised oxygen (minutes) | Reason for withdrawal |
| --- | --- | --- | --- | --- | --- |
| 1 Withdrawn from the trial | 31-Oct-14 | 13:25 | Cold humidified O_2_ | 5 | Cold humidifier not working. |
| 2 Withdrawn from the trial | 04-Mar-15 | 17:50 | Standard O_2_ therapy | 240 | Had associated wheeze and increased work of breathing, was not admitted with clinical symptoms of asthma or viral induced wheeze. |
| 3 Withdrawn from the trial | 28-Jun-15 | 14:30 | Heated humidified O_2_ | 0 | Father did not want child to participate |
| 4 Withdrawn from the trial | 20-Jun-14 | 13:25 | Standard O_2_ therapy | 58 | Deterioration in condition (planned admission PICU). |
| 5 Discontinuation from treatment | 31-Oct-14 | 13:25 | Cold humidified O_2_ | 5 | Stopped to allow switch to alternative O_2_ therapy |
| 6 Discontinuation from treatment | 18-Jan-16 | 12:15 | Heated humidified O_2_ | 260 | Stopped to allow switch to alternative O_2_ therapy |
| 7 Discontinuation from treatment | 04-Sep-14 | 15:58 | Standard O_2_ therapy | 291 | Stopped to allow switch to alternative O_2_ therapy |
| 8 Discontinuation from treatment | 03-Dec-14 | 18:45 | Heated humidified O_2_ | 90 | Stopped to allow switch to alternative O_2_ therapy |
| 9 Discontinuation from treatment | 17-Feb-15 | 15:30 | Heated humidified O_2_ | 985 | Stopped to allow switch to alternative O_2_ therapy |
| 10 Discontinuation from treatment | 04-Mar-15 | 17:50 | Standard O_2_ therapy | 240 | Stopped to allow switch to alternative O_2_ therapy |
| 11 Discontinuation from treatment | 12-Oct-15 | 08:40 | Heated humidified O_2_ | 895 | Stopped to allow switch to alternative O_2_ therapy |
| 12 Discontinuation from treatment | 20-Dec-15 | 03:05 | Cold humidified O_2_ | 255 | Stopped to allow switch to alternative O_2_ therapy |
| 13 Discontinuation from treatment | 03-Mar-16 | 03:00 | Cold humidified O_2_ | 48 | Stopped to allow switch to alternative O_2_ therapy |
| 14 Discontinuation from treatment | 23-Mar-15 | 17:10 | Cold humidified O_2_ | 635 | Stopped to allow switch to alternative O_2_ therapy |
| 15 Discontinuation from treatment | 01-Jul-15 | 11:30 | Heated humidified O_2_ | 545 | Stopped to allow switch to alternative O_2_ therapy |
| 16 Discontinuation from treatment | 26-Nov-15 | 13:01 | Heated humidified O_2_ | 477 | Stopped to allow switch to alternative O_2_ therapy |
| 17 Discontinuation from treatment | 12-Jul-16 | 23:50 | Heated humidified O_2_ | 180 | Stopped to allow switch to alternative O_2_ therapy |
| 18 Discontinuation from treatment | 01-Mar-16 | 13:15 | Heated humidified O_2_ | 52 | Stopped to allow switch to alternative O_2_ therapy |
