# Supplementary material for: Humidified and standard oxygen therapy in acute severe asthma in children (HUMOX): A pilot randomised controlled trial
Source: PLoS One. 2022 Feb 3;17(2):e0263044. doi: 10.1371/journal.pone.0263044 (PMC8812987; doi:10.1371/journal.pone.0263044)
Supplement: S1 File — (PDF) [file pone.0263044.s007.pdf]

“The role of heated humidified oxygen in asthma”
HUMOX Study

Chief Investigator: Dr Paul McNamara
Alder Hey Children’s Hospital, Liverpool

PARENT/GUARDIAN INFORMED CONSENT FORM

Name of child: _________________________

Participation Identification Number for this study: _________

Please initial box to confirm consent

1. I confirm that I have read and understand the information sheet(s) dated 23 October 2013 (version 2.0) for the above study and have had the opportunity to ask questions. ☐
2. I understand that my/my child’s participation is voluntary and that I am/he/she is free to withdraw at any time, without giving any reason, without my/his/her medical care or legal rights being affected. ☐
3. I understand that sections of any of my child’s medical notes may be looked at by individuals responsible for the study, monitors, auditors and sponsor representatives, where it is relevant to my child’s taking part in research. I give permission for these individuals to have access to my child’s records. ☐
4. I agree/agree for my child to take part in the Humox study. ☐

Signed: Date:

Name (print): Relationship to child:

Assent of Child:

I have had the opportunity to speak to the doctor or nurse and I am happy to take part in this study

Signed: Date:

Name of person taking consent:

Signed: Date:
