# Supplementary material for: Humidified and standard oxygen therapy in acute severe asthma in children (HUMOX): A pilot randomised controlled trial
Source: PLoS One. 2022 Feb 3;17(2):e0263044. doi: 10.1371/journal.pone.0263044 (PMC8812987; doi:10.1371/journal.pone.0263044)
Supplement: S2 File — (PDF) [file pone.0263044.s008.pdf]

"Does warm air help to improve your asthma?"
HUMOX Study

(For children aged 6-11 attending A&E with acute Asthma)

Version 2.0 Dated 23 Oct 2013

What is the study about?
Airways are small tubes in the chest that help you to breathe. When you have asthma the muscles in the airways tighten, making it hard to breathe.

We want to see whether giving warm air with added water makes it easier to breathe in an asthma attack.

Why have I been chosen?
You have been chosen because you've got asthma or wheeze and need oxygen.

What will happen if I agree to take part?
You will be given warm oxygen that is bubbled through water, cold oxygen that is bubbled through water or cold dry oxygen, by a face-mask that is put over your nose and mouth.

Are there any disadvantages to taking part in this study?
No

Will joining help me?
We hope it will help children with acute asthma in the future.

Do I have to take part?
No. It is up to you.

Will my information be kept secret?
Yes, all the information about you will be kept private. The study results will be available to anyone who wants to look at them but it won't be possible to tell who you are.

What if I don't want to do it anymore?
If you want to stop just tell your parents, doctor or a nurse. You can stop at anytime without giving a reason**.**

If you have any further questions they can be discussed with Paul McNamara (Chest doctor), Vanessa Compton (Physiotherapist) or Janet Clark (Research Nurse).
