# Supplementary material for: Humidified and standard oxygen therapy in acute severe asthma in children (HUMOX): A pilot randomised controlled trial
Source: PLoS One. 2022 Feb 3;17(2):e0263044. doi: 10.1371/journal.pone.0263044 (PMC8812987; doi:10.1371/journal.pone.0263044)
Supplement: S3 File — (PDF) [file pone.0263044.s009.pdf]

"Does warm oxygen instead of cold oxygen improve your asthma quicker?"
HUMOX Study

Patient Information Sheet
(For children aged 12-16 attending A&E with acute Asthma)

Version 2.0 Dated 23 Oct 2013

We are asking if you would like to take part in a research study to help to find the answer to the question, "Does warm oxygen instead of cold oxygen improve your asthma quicker?" Before deciding whether or not you wish to take part it is important for you to understand why the research is being done and what it involves. Please take the time to read this leaflet carefully. Ask us if there is anything that is not clear or if you would like more information. Talk about it with your family, friends, doctor or nurse if you want to.

What is the study about?
Normally, our airways add water to the air that we breathe. This makes it easier to keep them open. In an acute asthma attack, the muscles in the airways tighten and the airway lining becomes inflamed and starts to swell. This causes them to narrow, making it difficult to breathe.

As part of your asthma treatment you are being given oxygen via a face-mask. In most hospitals around the country, oxygen comes out of the wall, cold and dry. We would like to find out whether adding water and warming the oxygen you receive reduces the amount of time children need oxygen for and how long they spend in hospital. Our aim is to ensure we are giving children with asthma the best care we can.

Why have I been chosen?
You have been chosen because you have come to A&E with acute asthma or wheeze and need oxygen.

What will happen if I agree to take part?
As part of your asthma treatment, you will be given oxygen. If you agree to take part in this study, the type of oxygen you receive (either cold/dry, cold/bubbled through water or warmed/bubbled through water) will be decided by chance.

Are there any disadvantages to taking part in this study?
There are no disadvantages to taking part. You will receive all the same care and medication as normal.

Are there any benefits to taking part in this study?
No, there are none, but we hope it will help children with acute asthma in the future

Do I have to take part?
No, it is up to you. You are free not to take part or to withdraw from the research at any time and without giving a reason. It will not affect the treatment you receive.

If you would like to take part, you will be given this information sheet to keep and you will be asked to sign a assent form. We will ask your parents or guardian to sign a consent form too.

Will my information be kept confidential?
Yes, all the information about you will be kept private. The study will be published but it won't be possible to identify individual children or parents from what is written. This study has been approved by the North West Liverpool East Research Ethics Committee.

If you would like further information about the study please contact Dr Paul McNamara or Janet Clark using the details given below.

Should you have any complaints please contact the PALS team using the contact details below.

You can either contact:

Dr Paul McNamara (Principal Investigator),
Institute of Child Health,

Alder Hey Children's Hospital,
Eaton Rd,
Liverpool, L12 2AP
0151 282 4531

Ms Janet Clark (Research Nurse),
Medicines for Children Local Research Network,
Alder Hey Children's Hospital,
Eaton Rd,
Liverpool
0151 252 5673

The Patient Advisory Liaison Service (PALS)
Alder Hey Children's Hospital
Eaton Rd,
Liverpool, L12 2AP
Tele: 0151 252 5161
Email: pals@alderhey.nhs.uk
