# Supplementary material for: Humidified and standard oxygen therapy in acute severe asthma in children (HUMOX): A pilot randomised controlled trial
Source: PLoS One. 2022 Feb 3;17(2):e0263044. doi: 10.1371/journal.pone.0263044 (PMC8812987; doi:10.1371/journal.pone.0263044)
Supplement: S4 File — (PDF) [file pone.0263044.s010.pdf]

STUDY PROTOCOL

Humox Study

A pilot study to assess whether humidified oxygen is more effective that standard oxygen therapy in treating children with acute severe asthma

Study Sponsor:
Alder Hey Children's NHS Foundation Trust
Eaton Road,
Liverpool,
L12 2AP
Tel: +44 (0)151 252 5570

Funder:
National Institute for Health Research (NIHR)
Research for Patient Benefit funding stream

Chief Investigator: Professor Paul McNamara

Protocol Version: 4.0
Date: 23/11/2015
REC Ref: 13/NW/0738
ISRCTN number: ISRCTN62616194

Protocol Approval
Authorised by Chief Investigator:

Signature: ______________________

Date: 2/2/2017

Professor Paul McNamara,
Paediatric Respiratory Consultant
Alder Hey Children's NHS Foundation Trust
Eaton Road
Liverpool
L12 2AP
Tel: +44 (0)151 252 4531
Email: mcnamp@liv.ac.uk

Authorised on behalf of the Sponsor:

Signature: ______________________

Date: 2/2/2017

Miss Lucy Cooper
Research Governance and Quality Assurance Lead
Alder Hey Children's NHS Foundation Trust
Eaton Road
Liverpool
L12 2AP
Tel: +44 (0)151 252 5570
Email: lucy.cooper@alderhey.nhs.uk

TABLE OF CONTENTS

1 PROTOCOL SUMMARY 5

2 BACKGROUND INFORMATION 8

2.1 INTRODUCTION & RATIONALE 8
2.2 OBJECTIVES 10

3 POTENTIAL RISKS AND BENEFITS 10

3.1 POTENTIAL RISKS 10
3.2 POTENTIAL BENEFITS 11

4 SELECTION OF CENTRES 11

5 TRIAL DESIGN 11

6 STUDY POPULATION 11

6.1 INCLUSION CRITERIA 11
6.2 EXCLUSION CRITERIA 12

7 PARTICIPANT WITHDRAWAL 12

8 ENROLMENT AND RANDOMISATION 13

8.1 SCREENING 13
8.2 RANDOMISATION 13
8.2.1 RANDOMISATION PROCESS 14
8.2.2 RANDOMISATION CRF 15
8.2.3 RANDOMISATION TREATMENT 15

9 TRIAL TREATMENTS 15

9.1 INTRODUCTION 15
9.2 STORAGE 15
9.3 ADMINISTRATION OF STUDY TREATMENTS 15
9.4 BLINDING 16
9.5 CONCOMITANT MEDICATIONS AND TREATMENTS 16
9.6 CO-ENROLMENT GUIDELINES 16

10 ASSESSMENTS AND PROCEDURES 16

10.1 SCHEDULE FOR FOLLOW-UP 16
10.2 PROCEDURES FOR ASSESSING EFFICACY 17
10.2.1 YUNG'S ASTHMA SEVERITY SCORE 17
10.3 PROCEDURES FOR ASSESSING SAFETY 17
10.4 OTHER ASSESSMENTS 17

11 STATISTICAL CONSIDERATIONS 18

11.1 METHOD OF RANDOMISATION 18

11.2 OUTCOME MEASURES PRIORITISATION AND CONSENSUS ... 18
11.3 SAMPLE SIZE ... 18
11.4 ANALYSIS PLAN ... 18

12 SAFETY ... 19

12.1 TERMS AND CONDITIONS ... 19
12.2 NOTES ON ADVERSE EVENT INCLUSIONS AND EXCLUSIONS ... 19
12.2.1 INCLUDE ... 20
12.2.2 DO NOT INCLUDE ... 20

13 ETHICAL CONSIDERATIONS ... 20

13.1 ETHICAL CONSIDERATIONS ... 20
13.2 ETHICAL APPROVAL ... 21
13.3 INFORMED CONSENT PROCESS ... 21

14 TRIAL MONITORING ... 21

14.1 SOURCE DOCUMENTS ... 21
14.2 DATA CAPTURE METHODS ... 22
14.2.1 CASE REPORT FORMS ... 22
14.3 DATA MONITORING AT MC CTU ... 22
14.4 CLINICAL SITE MONITORING ... 22
14.5 CENTRAL MONITORING ... 23
14.6 CONFIDENTIALITY ... 23
14.7 RECORDS RETENTION ... 23

15 INDEMNITY ... 24

16 FINANCIAL ARRANGEMENTS ... 24

17 TRIAL COMMITTEES ... 24

17.1 TRIAL MANAGEMENT GROUP (TMG) ... 24
17.2 TRIAL STEERING GROUP (TSC) ... 24

18 PUBLICATION ... 24

19 APPENDICES ... 25

19.1 APPENDIX 1: GLOSSARY ... 25
19.2 APPENDIX 2: TRIAL MANAGEMENT GROUP ... 26
19.3 APPENDIX 3: TRIAL STEERING GROUP ... 26

1 Protocol Summary

Title:

A pilot study to assess whether humidified oxygen is more effective than standard oxygen therapy in treating children with acute severe asthma

Population:

Children/adolescents between 2-16 years of age attending A&E with a clinical diagnosis of acute severe or potentially life-threatening asthma according to BTS/SIGN guidelines and who are still requiring oxygen after initial standard nebuliser therapy.

Study Centres:

Alder Hey Children's NHS Foundation Trust

University Hospitals of Morcambe Bay NHS Foundation Trust

Countess of Chester Hospital NHS Foundation Trust

Warrington & Halton Hospitals NHS Foundation Trust.

Study Duration:

Total study duration for each patient will be the time that individual requires oxygen in hospital because of his or her asthma. Patients will be screened at presentation to A&E and if eligible (i.e. oxygen saturations below 92%), their parents/guardian will be provided with information about the study. Treatment will then be initiated according to the BTS guidelines. Standard oxygen therapy will be administered according to local practice and three back-to-back combined salbutamol/ipratropium bromide nebulisers given. If the patient still has oxygen saturation levels <92% following this initial management (typically approximately 30 minutes after presentation to A&E, but potentially up to 90 minutes), consent to participate in the study will be sought from parents/guardians. Oxygen treatment will then be changed (or not) according to randomisation. Trial assessments for the most part will reflect those routinely performed in this patient population and will be completed at randomisation, 2, 4, 6, 8, 12 hours following randomization, and then every 6 hours for as long as the patient needs oxygen. A questionnaire determining parental views on what they consider are meaningful outcome measures for studies on acute asthma will be given to parents (and where appropriate patients) during their stay in hospital. Another questionnaire assessing respiratory symptoms will be given to participants parents/guardians at a routine follow-up outpatient clinic appointment organized by the patients clinical team three months following discharge from hospital or posted to families if no such appointment is arranged.

Description of Intervention(s):

In the A&E department, each child will be randomized to receive one of the following three treatments:

1. *Heated Humidified Oxygen:* this will be delivered by a Fisher & Paykel MR850 humidifier and a RT408 Oxygen Therapy System through a

System face-mask (No 1120 or 1100 depending on patient size). The humidifier will be set to a temperature of 31 ^{0}C and the percentage inspired oxygen titrated to maintain the patient’s oxygen saturations above 92%. The humidifier will be filled with sterile water and the levels monitored and topped up as necessary.

2. *Cold, humidified Oxygen:* this will be given through an inter-surgical humidifier nebuliser, inserted into a bottle of sterile water and attached to wall mounted low flow oxygen. Elephant tubing will be used to connect the nebuliser device to the patient's face-mask. Up to 60% oxygen will be titrated to maintain the patient's oxygen saturations above 92%. If the patient requires more than 60% oxygen, a Rusch multifit nebuliser with BOC adapter will be used in the same way.
3. *Standard Oxygen therapy:* Cold (15^{0}C), dry (un-humidified) oxygen directly from the wall at the patient bedside will be given in A&E via a Non rebreather mask. Once the patient requires less than 10 L O2 (approx 50 %) they may be changed to nasal cannula. Less than 10 L via a NRB will result in poorer CO2 clearance.

Upon transfer to in-patient ward, children will continue to receive the same treatment they were randomised to in A&E until they stop needing oxygen.

Objectives:

This external pilot open randomised controlled trial will generate data examining study feasibility and specifically data on accrual, adherence and outcome measure stability. Data collected will inform power calculations for a future NIHR HTA multi-centre randomised controlled trial of humidified oxygen in acute severe asthma. The aim of this subsequent definitive study will be to determine whether humidified oxygen (either heated or non-heated) is more effective than standard cold, dry oxygen therapy, in the treatment of children with severe and potentially life threatening asthma.

Thirty children with acute severe asthma will be recruited into each treatment arm (90 in total) and the following outcome measures will be examined:

- Length of time continuously spent in oxygen
- Time until treatment ‘stepped down’ to hourly, two-hourly and four-hourly nebulised therapy
- Differences in oxygen saturation in air at set time points after entry into the study
- Changes in *Yung’s Asthma Severity Score*
- Number of Salbutamol and Ipratropium Bromide nebules required by each patient following randomisation
- Requirement for escalation of treatment (i.e. need for intravenous salbutamol/aminophylline or HDU/PICU)
- Adverse events/tolerability
- Length of stay in hospital

Schematic Representation of Study Design:

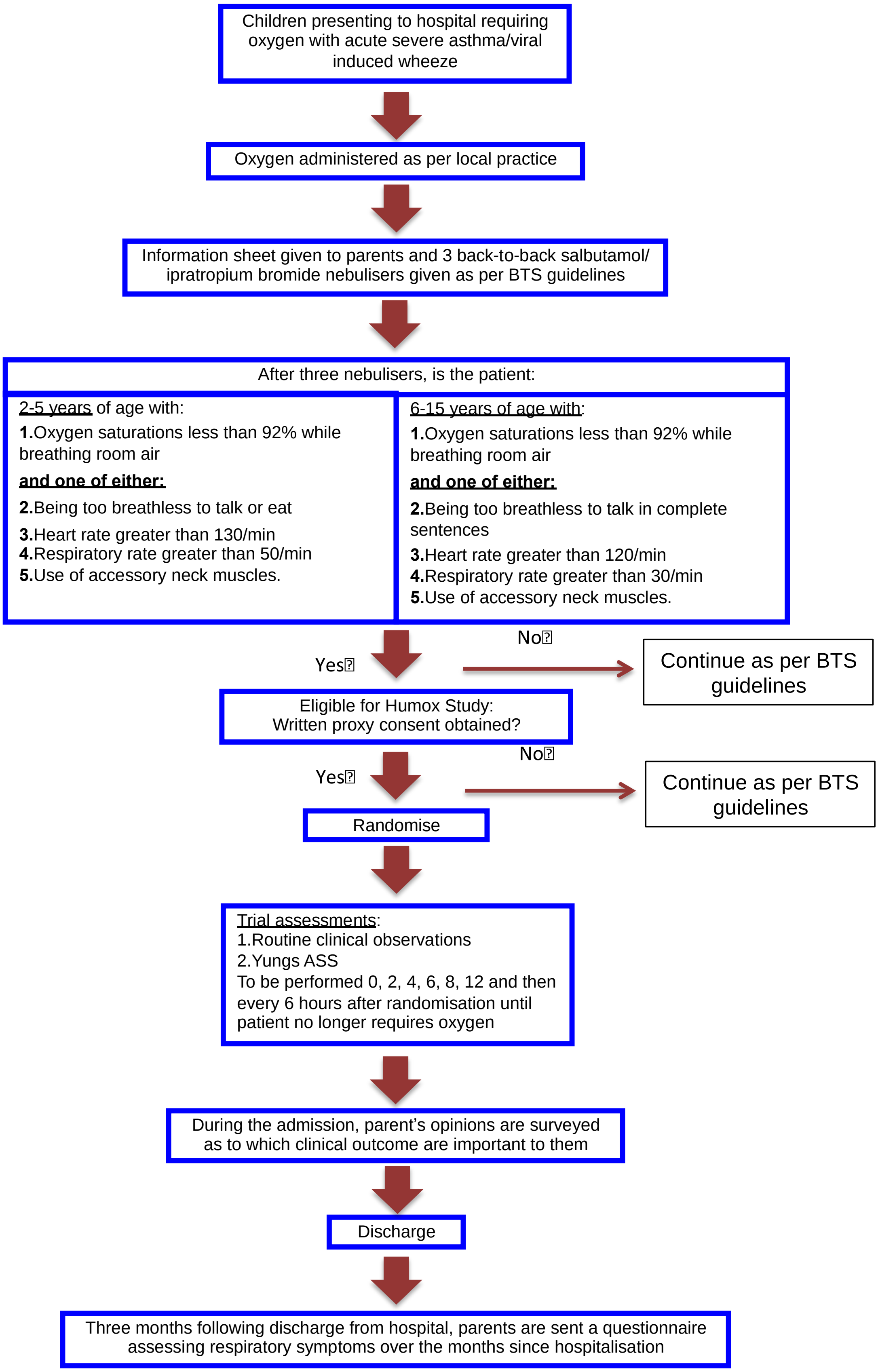

2 Background information

2.1 Introduction & Rationale

Asthma is the most common chronic disease of childhood and worldwide has dramatically increased in prevalence over the last 30 years[1]. In the UK, there is a person with asthma in one in five households and 1.1 million children are currently receiving treatment for this condition. In Liverpool we have a particular problem: Alder Hey admits one in thirty of all children hospitalised with acute severe asthma in the UK (approximately 1000/annum).

Airway mucus hyper-secretion is a cardinal feature of asthma. Mucus accumulates in the airways leading to obstruction, low oxygen levels and potentially, respiratory failure. Mucus and other inhaled particulate matter is normally moved from the lower to the upper airways by the mucociliary transport system (MCTS)[2]. Microscopic hairs on epithelial lining cells move secretions upward until they reach the throat where they are swallowed.

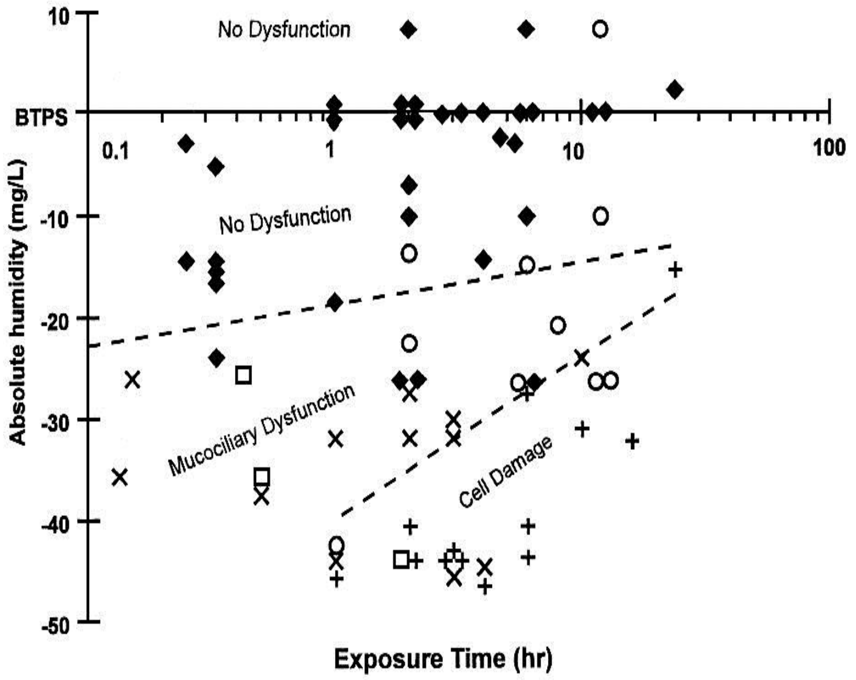

Figure 1 *A humidity and exposure map produced from published data series in dogs. Each point represents a single measurement of absolute humidity (derived from air temperature and relative humidity) and time of exposure in hours. The dysfunction state observed at each measurement is coded (diamonds, no dysfunction; circles, mucus thick or thin; squares, mucociliary transport stopped; times signs, cilia stopped; plus signs, cell damage). Boundaries between dysfunction categories have been added based on results.*

The MCTS is at its most efficient if inspired air is humidified and at body temperature by the time it reaches the carina (the bifurcation of the bronchi)[4;5]. Small changes in humidity or temperature either way cause profound effects on mucosal function, with the amount of dysfunction and damage dependent on the extent of deviation from normal and the time of exposure **(Figure 1)**[3]. Thus, mucociliary dysfunction occurs within 10 minutes of exposure to dry (unhumidified) air and after 3-4 hours significant epithelial damage is seen which continues to worsen with time. The highest dysfunction scores are

found in airways exposed to dry air at temperatures between 15-24^{0}C **(Figure 2)**.

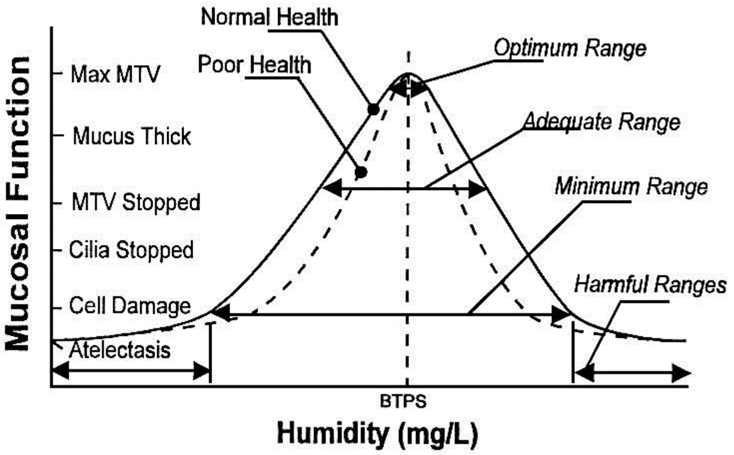

Figure 2: *Williams et al proposed a model of the relationship between inspired gas humidity and temperature, exposure time and mucosal function based on the data in* ***Figure 1***[6]*. This figure shows how the mucociliary transport system can be affected by altering the humidity levels and how that effect is increased during poor health or respiratory illness.*

It is for these reasons that heated, humidified oxygen is routinely given on intensive care. Studies in intubated adults and children have shown that maintaining airway temperature close to body temperature beneficially affects lung function, mucus clearance and in NIV, patient compliance.

With portable humidification devices becoming more widely available, it is now possible to give heated humidified oxygen in acute emergency situations and on inpatient medical wards. To date only one study has examined its effect in acute asthma. This was a study in children (6-17yrs) whose initial nebulised bronchodilator therapy only was driven by humidified oxygen, either at room temperature or at 40^{0}C. This short intervention was well tolerated and peak flows increased more following nebulisation with heated humidified oxygen than with humidified oxygen at room temperature. Overall however, there is a dearth of evidence to support its use in either children or adults, a situation recognised by both the *British Thoracic Society (BTS) Guidelines for Emergency Oxygen Use* (2008) and the 2012 update of the *BTS Guideline on the Management of Asthma*.

Currently in the UK, children (and adults) with acute severe asthma, receive cold (15^{0}C), dry (un-humidified) oxygen in the A&E department for between one and four hours prior to transfer to the ward. If admitted, they may or may not receive humidified oxygen depending on their local hospital asthma guidelines. Through the National Paediatric Respiratory Nurses Group, we surveyed the use of humidified oxygen nationwide, obtaining responses from 17 paediatric departments from Exeter to Melrose. No hospital gave humidified oxygen in A&E and over half didn't give humidified oxygen following admission to medical wards. Of those that gave humidified oxygen, approximately a quarter could also give warmed humidified oxygen. All

surveyed felt that any study on the use of humidified oxygen in children with acute severe asthma would be worthwhile.

2.2 Objectives

The overall aim of this study is to determine whether humidified oxygen (either heated or non-heated) is more effective than standard oxygen therapy in the treatment of children with severe and potentially life threatening asthma.

This pilot study will determine the most appropriate outcome measures for a planned large definitive multi-centre study, hopefully funded by the HTA. The potential impact of any definitive study could be profound, having the potential to change the way we manage acute asthma in children and encouraging uniformity of treatment, so obviously absent in our nationwide survey of current practices.

The following outcome measures will be examined:

- Length of time in oxygen
- Time until treatment 'stepped down' to hourly, two-hourly and four-hourly nebulised therapy
- Differences in oxygen saturation in air at set time points after entry into the study
- Changes in *Yung's Asthma Severity*
- Number of Salbutamol and Ipratropium Bromide nebules required by each patient following randomisation
- Requirement for escalation of treatment (i.e. need for intravenous salbutamol/aminophylline or HDU/PICU)
- Adverse events/tolerability
- Length of stay in hospital

A questionnaire determining parental views on what they consider are meaningful outcome measures for studies on acute asthma will be given to parents (and where appropriate patients) during their stay in hospital.

All patients will be followed up in paediatric outpatient clinics, 2-4 months following admission to hospital. At this appointment, parents/guardians will be given the two-page Liverpool respiratory symptoms questionnaire to complete by the research nurse, to assess symptoms over the three-month period following discharge from hospital. If the patient fails to attend clinic, then the questionnaire will be sent to their parents. If no questionnaire is returned within three weeks, parents will be contacted and asked whether they would like to complete the questionnaire over the phone.

3 Potential Risks and Benefits

3.1 Potential Risks

At present, all three treatment interventions are currently being given routinely on medical wards throughout the UK and around the world. We are

not aware of any known significant risks associated with any of the treatments.

3.2 Potential Benefits

The potential benefit associated with this study is to provide some much needed guidance about how best to manage acute severe asthma in children, thereby hopefully shortening lengths of stay in hospital and potentially need for HDU/PICU.

4 Selection of centres

To provide pilot data on ease of recruitment in both secondary and tertiary care, one large paediatric teaching hospital (Alder Hey) and three district general hospitals (Royal Lancaster Infirmary, Warrington and Halton, and Countess of Chester) have been selected.

For the planned definitive study, it is envisaged that secondary and tertiary centres throughout the UK will be invited to participate with each participating center (and investigator) identified on the basis of:

- An institution with provision for emergency treatment of children and young people with acute asthma symptoms
- Having at least one lead clinician with specific interest in and responsibility for supervising and managing children who present with acute exacerbations of asthma
- Showing enthusiasm to participate in the study
- Ensuring that sufficient time, staff and adequate facilities are available for the trial
- Providing information to all supporting staff members involved with the trial or with other elements of the patients management
- Identifying that they will be able to recruit a specified target number of patients
- Acknowledging and agreeing to conform to the administrative and ethical requirements and responsibilities of the study, including signing up to Good Clinical Practice and other regulatory documentation

5 Trial Design

This is an external multi-centre, open randomised controlled pilot study which compares the efficacy of heated humidified oxygen to the standard therapy of cold dry oxygen in the management of children admitted to hospital with acute severe asthma.

6 Study Population

6.1 Inclusion Criteria

Patients between 2-16 years of age attending A&E with a clinical diagnosis of acute severe or potentially life-threatening asthma according to the

BTS/SIGN guidelines and who are still requiring oxygen after initial standard nebuliser therapy.

For children 6 years and older severe asthma will be based on the following criteria being met:

1. Oxygen saturations less than 92% while breathing room air
   and one of either:
2. Being too breathless to talk in complete sentences
3. Heart rate greater than 120/min
4. Respiratory rate greater than 30/min
5. Use of accessory neck muscles.

For children aged 2-5 years of age, severe asthma will be based on the following criteria being met:

1. Oxygen saturations less than 92% while breathing room air
   and one of either:
2. Being too breathless to talk or eat
3. Heart rate greater than 130/min
4. Respiratory rate greater than 50/min
5. Use of accessory neck muscles.

6.2 Exclusion Criteria

The following are exclusion criteria for participation in the study:

- Requiring admission to intensive care at time of recruitment
- Previous participation in this iteration of the Humox study
- Other significant respiratory disease (chronic lung disease of prematurity, previous significant chest infections)
- Any other significant underlying medical problem (immunodeficiency, neurological and cardiac conditions)
- Previously or currently involved with a trial of a medicinal product in the three months preceding screening
- Parents/guardians who are unable to give informed consent

7 Participant Withdrawal

In consenting to the trial, patients (and their parents) are consented to trial intervention, follow-up and data collection. If voluntary withdrawal occurs, the patient (or parent/legal representative) should be asked to allow continuation of scheduled evaluations, complete and end-of-study evaluation if appropriate, and be given appropriate care under clinical supervision until the symptoms of any adverse event resolve or the patient's condition becomes stable.

Patients are free to withdraw consent at any time without providing a reason. Patients who wish to withdraw consent for the trial will have anonymised data collected up to the point of that withdrawal of consent included in the analyses. The patient will not contribute further data to the study and the MC CTU should be informed and a withdrawal CRF should be completed. Data

up to the time of withdrawal will be included in the analyses unless the patient explicitly states that this is not their wish.

Patients requiring intubation and ventilation on Intensive Care will be withdrawn but will continue to be followed up for outcome data, unless parents/guardians expressly request otherwise. Failure of compliance to treatment is covered in Section 13.

8 Enrolment and Randomisation

The target population will be children and adolescents attending A&E department with an acute severe asthma exacerbation and who require oxygen as part of their routine supportive care after initial standard nebuliser treatment.

8.1 Screening

All patients aged 2-16 years with severe/life-threatening asthma, will be screened to identify potentially eligible participants for the study. Due to the requirement to provide prompt treatment in an emergency setting, patient information and consent forms will be provided to the parent or legally acceptable representative concurrently to screening assessments taking place.

The following will be recorded on the CRF as part of a screening assessment at triage:

- Confirmation that the child is aged 2-16 years
- Assessment of asthma severity (based on age-specific BTS guidelines)
- SaO_2, Oxygen therapy (L/min) and Respiratory Rate
- Yung's Asthma Severity Score (incorporating wheeze, accessory muscle use and heart rate)
- Collection of demographic information including:
    - Age of asthma onset
    - Current asthma medication
    - Duration of current exacerbation
    - Number of previous hospital admissions to asthma (including the number of previous admissions that resulted in PICU)
    - History of food allergy, hayfever and eczema

Contemporaneously with the screening phase, patients will receive three 'rounds' of nebulised treatment with salbutamol and ipratropium bromide. This will be recorded on the CRF. Any other medication given (such as oral steroids or treatment given during transport to hospital) will also be documented.

8.2 Randomisation

After completion of the screening phase and initial nebulised treatment, the patient will be re-assessed. If they require oxygen and fulfill one of the age-specific criteria listed above, parents/guardians will be asked for consent for

their child to participate in the study. Patients no longer meeting the criteria will be excluded and will continue to be treated as per standard hospital practice.

Participants will be randomised once:

- Eligibility criteria have been fulfilled
- Fully informed written proxy consent has been obtained
- Screening assessments have been completed

8.2.1 Randomisation Process

Randomisation will be carried out by a designated staff member (as specified on the delegation log) if the patient has oxygen saturation levels <92% following initial management and consent has been sought. Randomisation should take place within 90 minutes of the first nebuliser treatment being given in hospital.

The allocated randomisation number and treatment type will be provided to centres in the form of a series of randomisation envelopes, similar to those used for payslips. The randomisation envelopes will be held in a designated area depending on site, and the randomising person will select the next sequentially numbered envelope depending on the age of the child. At randomisation, participants will be allocated a unique randomisation number and allocation. Trial entry and randomisation number should be recorded in the patient's medical notes.

The randomisation envelopes cannot be viewed without fully opening and their construction is resistant to accidental damage or tampering.

Once the information has been completed on the randomisation envelopes the pages should be split and processed as follows:

Page 1 & 2 of the randomisation envelope containing information on the allocation should be completed and returned to the MC CTU in the pre-addressed envelope
Page 3 is placed back in the randomisation folder in the appropriate section, ensure date and time of randomisation are recorded on the CRF

The research nurse will check to ensure that the correct number of randomisation envelopes is present, that they are intact and that the sequential numbering system is maintained. If any randomisation envelopes are damaged, opened accidentally, mislaid or there are discrepancies this should be to the lead RN at Alder Hey who will record any errors and report to the MC CTU immediately.

If a randomisation envelope is opened for the wrong age the researcher should continue with the allocated treatment, but report the incidence to the lead RN at Alder Hey.

8.2.2 Randomisation CRF

At randomisation, the following data will be recorded in the Randomisation Assessment part of the CRF:

- Time of Randomisation
- SaO_2, Oxygen therapy (L/min), Temperature and Respiratory Rate.
- Asthma Severity Score (incorporating wheeze, accessory muscle use and heart rate)

8.2.3 Randomisation Treatment

Trial treatment will begin as soon as possible after the initial nebuliser treatment has concluded and assessments have been performed. The clinician/nurse should ensure that the duration between obtaining consent, performing assessments and the start of trial treatment does not impact on the well-being of the participant. Trial equipment (humidifiers etc) will be located in a designated area within the A&E department, accessible to staff 24 hours a day. Details of randomised patients should be entered on the randomisation log kept with the TMF/ISF.

9 Trial Treatments

9.1 Introduction

This RCT compares three methods of administering oxygen to patients with asthma. Patients will be randomised to warm humidified, cold humidified or cold dry oxygen in a ratio 1:1:1.

9.2 Storage

Humidifiers/equipment will be stored in an area designated by the lead clinician/research nurse at each individual site, following discussions with A&E/acute assessment unit staff.

9.3 Administration of Study Treatments

In the A&E department, each child will be randomized to receive one of the following three treatments:

1. *Heated Humidified Oxygen:* this will be delivered by a Fisher & Paykel MR850 humidifier and a RT408 Oxygen Therapy System through a System face-mask (No 1120 or 1100 depending on patient size). The humidifier will be set to a temperature of 31 ^{0}C and the percentage inspired oxygen titrated to maintain the patient's oxygen saturations above 92%. The humidifier will be filled with sterile water and the levels monitored and topped up as necessary..
2. *Cold, humidified Oxygen:* this will be given through an inter-surgical humidifier nebuliser, inserted into a bottle of sterile water and attached to wall mounted low flow oxygen. Elephant tubing will be used to connect the nebuliser device to the patient's face-mask. Up to 60% oxygen will be titrated to maintain the patient's oxygen saturations above 92%. If the patient requires more than 60% oxygen, a Rusch multi-fit nebuliser with BOC adapter will be used in the same way.

3. *Standard Oxygen therapy:* Cold (15^0C), dry (un-humidified) oxygen directly from the wall at the patient bedside will be given in A&E via a non-rebreather mask. Once the patient requires less than 10L O2 (approximately 50% FiO_2), they may be changed to nasal cannula.

Upon transfer to in-patient ward, children will continue to receive the same treatment they were randomised to in A&E until they stop needing oxygen.

9.4 Blinding

The study will compare three different ways of administering oxygen. As such, it will not be possible for the person administering the intervention to be 'blinded'.

9.5 Concomitant Medications and Treatments

Additional medications used to treat exacerbations of the patient's condition will be recorded on a separate bronchodilator/steroids form.. No medications are contra-indicated.

9.6 Co-enrolment Guidelines

To avoid potential confounding issues, patients should not be recruited to other trials concerning the acute management of asthma. Where recruitment to another trial is considered to be appropriate and without having any detrimental effect on the Humox trial, this must first be discussed with the Chief Investigator or delegated other.

10 Assessments and Procedures

10.1 Schedule for Follow-up

Following randomisation, trial participants will be assessed at set time intervals for as long as they require oxygen and until discharge.

i. 2, 4, 6, 8, 12 hours and then every 6 hours following start of intervention
   a. Completion of ASS
   b. Respiratory rate, $Sa0_2$ in air (wait 2 minutes for saturations to stabilise), Oxygen therapy (L/min)
ii. Before discharge:
   a. Completion by parent/guardian (and patient if appropriate) of a questionnaire assessing views on what they consider are meaningful outcome measures for studies on acute asthma
iii. Three months following discharge, parents/guardians will be asked about respiratory symptoms since discharge using a validated respiratory symptom questionnaire. If the child is booked into an outpatient clinic approximately three months following admission, they will be given the questionnaire then. If this is not so, then a questionnaire will be sent to them by post with a stamped addressed envelope. If the questionnaire hasn't been returned within three weeks, parents will be reminded by telephone and asked if they would prefer to complete the questionnaire over the phone.

10.2 Procedures for Assessing Efficacy

Asthma severity will be assessed as follows:

10.2.1 Yung's Asthma Severity Score

Yung's asthma severity score will be assessed at regular 2hrly time intervals initially, then 4 and 6 hrly as described above. This score has been validated as a measure of asthma severity in children and has been demonstrated to be reproducible and reliable with good inter-observer agreement and correlates well with oxygen saturations and lung function[1]. It has recently been used in a NIHR study of nebulised magnesium sulphate in the treatment of acute severe asthma (MAGNETIC).

1.Yung M. Evaluation of an asthma severity score. JPCH 1996; 32:261-264

10.3 Procedures for Assessing Safety

Adverse events (AEs) (see section 12.1-2) will only be reported for participants where consent has been obtained and the causal relationship to the trial treatment (oxygen or delivery equipment) has been assessed and judged by the investigator to be related to the trial treatment, which occurs from the start of randomised treatment until final study assessment. The events should be recorded on a Related Adverse Event Form, which should be transmitted to the CTU within seven days of the clinical research team becoming aware of the event.

All related AEs should be followed until satisfactory resolution or until the investigator responsible for the care of the participant deems the patient to be stable.

Follow-up information for an AE should be noted on the related AE form under the heading marked 'outcome'.
When reporting AEs the investigator responsible for the care of the participant should apply the following criteria to provide information relating to event outcomes: resolved; resolved with sequelae (specifying with additional narrative); not resolved/ongoing; ongoing at final follow-up; unknown.

There are no serious adverse events anticipated which are related to the study interventions.

10.4 Other Assessments

The source of data will be case report forms patient notes, prescription charts and hospital computer systems. The need for oxygen will be assessed every hour with inspired oxygen titrated to maintain the patient's saturations greater than 93%. The time taken for patients to improve based on the 'stepping down' of their nebulised therapy (i.e. changed to hourly, 2 hourly or 4 hourly nebulisers) will be recorded. ASS observations will cease when the patient no longer requires the intervention i.e. the patient no longer requires oxygen. Assessment of fitness for discharge will be at baseline, then 6 hourly until "fit for discharge" criteria are met, with each site using its own paediatric asthma pathway to assess when this has happened.

11 Statistical Considerations

11.1 Method of Randomisation

The randomisation code list will be generated by a statistician (who is not involved with the HUMOX study) at the MC CTU. Participants will be randomised using two sets of randomisation envelopes, (one set for 2-5 year old children, and the other for 6-16 year olds). Participants will be randomised to warm humidified, cold humidified or cold dry oxygen in a ration 1:1:1.

11.2 Outcome Measures Prioritisation and Consensus

The following outcome measures will be examined in this study:

- Length of time in oxygen
- Time until treatment 'stepped down' to hourly, two-hourly and four-hourly nebulised therapy
- Yung's asthma severity score
- Number of Salbutamol and Ipratropium Bromide nebules required by each patient
- Requirement for escalation of treatment (i.e intravenous Salbutamol/Aminophylline or HDU/PICU)
- Adverse events/tolerability
- Length of stay in hospital

These outcomes have been identified as relevant and important in a previous exercise involving consumers and paediatricians (MCRN Respiratory and General Paediatrics Clinical Studies Groups), however their relative importance has not been assessed. A consensus exercise will be undertaken to establish which outcome(s) will be taken forward to the definitive randomised controlled trial. This will involve: (1) trial participants/families (research nurses will give a one-page questionnaire to parents/guardian once the patient has become stable and has come out of oxygen), (2) lay members of the study Steering Group, (3) the MC Young Person's Advisory Group, (4) Asthma UK, (5) MC Respiratory and General Paediatric Clinical Studies Groups.

In addition, outcome measure stability, feasibility of data collection and missing data events from the pilot study will contribute to selection of the most appropriate outcome(s) based on both consensus and feasibility.

11.3 Sample size

This is an external pilot RCT. A pragmatic sample size of 30 in each intervention arm has been set following statistical advice.

11.4 Analysis Plan

To ensure that the enrolled sample is representative of the target population demographic and clinical data will be initially described using summary statistics, means, medians, standard deviations etc, as appropriate. To determine the balance of the treatment/control groups, in terms of demographic and clinical variables, standard hypothesis tests and analysis of variance will be undertaken. As this is a pilot study and consequently not powered to detect a significant difference between groups in the main

outcome measures, no formal hypothesis tests or interim analysis are planned. The main outcome measures will be presented using summary statistics and, as an aid to identifying potential outcome measures for a definitive trial, the proportion of missing values will be assessed. To assess the feasibility of undertaking a definitive trial accrual and drop-out rates will be assessed using proportions and graphical representations.

12 Safety

12.1 Terms and Conditions

Adverse Event (AE)

Any untoward medical occurrence in a subject to whom a medicinal product has been administered, including occurrences which are not necessarily caused by or related to that product.

Serious Adverse Event (SAE)

Any adverse event, that:

- results in death
- is life-threatening* (subject at immediate risk of death)
- requires in-patient hospitalisation or prolongation of existing hospitalisation**
- results in persistent or significant disability or incapacity, or
- consists of a congenital anomaly or birth defect
- is an other important medical event that may jeopardise the subject***

*'life-threatening' in the definition of 'serious' refers to an event in which the patient was at risk of death at the time of the event; it does not refer to an event which hypothetically might have caused death if it were more severe.

**Hospitalisation is defined as an inpatient admission, regardless of length of stay, even if the hospitalisation is a precautionary measure for continued observation. Hospitalisations for a pre-existing condition, including elective procedures that have not worsened, do not constitute an SAE.

***Other important medical events that may not result in death, be life-threatening, or require hospitalisation may be considered a serious adverse event/experience when, based upon appropriate medical judgment, they may jeopardise the subject and may require medical or surgical intervention to prevent one of the outcomes listed in this definition.

12.2 Notes on Adverse Event Inclusions and Exclusions

Only related and unexpected adverse events/ severe adverse events will be reported. For the purposes of this study, a related event is one related to the intervention/therapy, not the underlying condition itself. For related and unexpected events:

12.2.1 Include

- Failure to tolerate oxygen therapy e.g. failing to tolerate face mask, heated humidity/facial sore
- Nose bleeds
- Incorrect set-up of equipment/equipment failure.

12.2.2 Do not include

- Medical or surgical procedures
- Pre-existing disease or conditions present before treatment that do not worsen
- Situations where an untoward medical occurrence has occurred
- The disease being treated or associated symptoms/signs unless more severe than that expected for the patient's condition
- Changes in the amount of oxygen administered immediately following change in route of oxygen administration

13 Ethical Considerations

13.1 Ethical considerations

This study will abide by the principles of the World Medical Association Declaration of Helsinki (1964) and the Tokyo (1975), Hong Kong (1989) and South Africa (1996) declarations.

We consider the specific ethical issues relating to participation in this trial to be:

- Limited time for consideration of trial entry; this trial is exploring the effects of humidified oxygen in the acute management of children with severe asthma, a condition requiring prompt intervention in A&E departments. Due to the very nature of the condition and intervention being investigated, parents are required to be informed about the trial and make a decision regarding entry within 90 minutes of beginning standard treatment. Recruiting investigators will be clinicians/nurse specialists experienced at imparting important information to parents in situations of extreme stress. Parents will be made aware of alternative treatments and of their right to withdraw the child from the child at any time without the child or family being subject to any resultant detriment.
- Informed consent in a paediatric population. The parent or legal representative of the child will have an interview with the investigator, or a designated member of the investigating team, during which they will be given the opportunity to understand the objectives, risks and inconveniences of the trial and the conditions under which it is to be conducted. They will be provided with written information and contact details of the local study personnel should they require further information. Simplified written information will be available for children 6-11 years, those aged 12-16 years and assent will be obtained when possible. The simplified sheets are broken into age groups as a guide only, and the researcher/clinician may provide the most appropriate

version at their discretion, taking into account individual child circumstances (consulting the parents if appropriate).

13.2 Ethical Approval

The trial protocol and all substantial amendments will be submitted for review to a Multi-centre Research Ethics Committee (MREC). As this study is an NIHR CRN Portfolio study, site specific assessment will take place as each site via CSP.

Proxy consent from the parent or legally acceptable representative should be obtained prior to each patient participating in the trial, after a full explanation has been given of the treatment options, including the conventional and generally accepted methods of treatment. Age and stage of development specific patient information and consent leaflets should also be implemented and patient assent obtained where appropriate. The right of the parent/legal representative to refuse consent for the minor to participate in the trial without giving reasons must be respected. After the patient has entered the trial, the clinician must remain free to give alternative treatment to that specified in the protocol, at any stage, in he/she feels it to be in the best interest of the patient. However the reason for doing so should be recorded and the patient will remain within the trial for the purpose of follow-up and data analysis. Similarly, the parent/legal representative of the patient remains free to withdraw the patient at any time from the protocol treatment and trial follow-up without giving reasons and without prejudicing the further treatment of the minor.

13.3 Informed Consent Process

Informed consent is a process initiated prior to an individual agreeing to participate in a trial and continues throughout the individual's participation. In obtaining and documenting informed consent, the investigator should comply with applicable regulatory requirements and should adhere to GCP and ethical principles.

Due to the nature of the study and the requirement to provide prompt treatment in an emergency setting, there will be a short window of 90 minutes available for obtaining consent in the A&E department/Paediatric Assessment Unit.

14 Trial Monitoring

Trial monitoring is carried out to ensure that the rights and well-being of human participants are protected during the course of a clinical trial. A risk assessment will be performed by the Sponsor to determine the level and type of monitoring required for specific hazards. This is a non-CTIMP study and the level and type of monitoring will be commensurate with this.

14.1 Source Documents

Source data*: All information in original records and certified copies of original records of clinical findings, observations, or other activities in a clinical trial necessary for the reconstruction and evaluation of the trial. Source data are contained in source documents (original records or certified copies). (ICH E6, 1.51).*

***Source documents**: Original documents, data, and records (e.g., hospital records, clinical and office charts, laboratory notes, memoranda, subjects diaries or evaluation checklists, pharmacy dispensing records, recorded data from automated instruments, copies or transcriptions certified after verification as being accurate copies, microfiches, photographic negatives, microfilm or magnetic media, x-rays, subject files, and records kept at the pharmacy, at the laboratories and at medico-technical departments involved in the clinical trial). (ICH E6, 1.52).*

The Sponsor will determine which data in the CRF do not constitute source data and for data the CRF is considered the source document (e.g. Yung's Asthma Severity Score assessments).

Trial monitoring will be conducted according to the Sponsor's standard operating procedure SOP 006 (Monitoring and auditing of research projects: general).

14.2 Data Capture Methods

14.2.1 Case Report Forms

The case report form (CRF) is the primary data collection instrument for the study. All data requested on the CRF must be recorded. All missing data must be explained. For the Yung's severity score, a box indicating 'Not Assessed' has been included for each domain at each time point on the CRF. If a space on the CRF is left blank because the procedure was not done or the question was not asked, write "N/D". If the item is not applicable to the individual case, write "N/A".

All entries should be printed legibly in black ink. If any entry error has been made, to correct such an error, draw a single straight line through the incorrect entry and enter the correct data above it. All such changes must be initialled and dated. DO NOT ERASE OR WHITE OUT ERRORS. For clarification of illegible or uncertain entries, print the clarification above the item, then initial and date it. Originals should be sent to the MC CTU and the copies securely retained at site.

14.3 Data Monitoring at MC CTU

Data stored at MC CTU will be checked for missing or unusual values (range checks) and checked for consistency within participants over time. Data queries will be generated as required and query forms will be sent to delegated individuals (nominated research nurse) at study sites. They will provide responses and return copies of the completed data query forms to MC CTU, where the appropriate corrections will be made on the study database.

14.4 Clinical Site Monitoring

Site monitoring may be deemed to be necessary as a result of central data checks by the Sponsor. In order to perform its role effectively, the Sponsor will delegate responsibility to the site research nurse and principal investigator for access to primary data. Checks will be conducted to ensure

copies of signed consent forms are held appropriately within patient records and site files.

14.5 Central Monitoring

Data submitted to the database will be centrally monitored by the CTU to ensure as far as possible that CRF data collected are consistent with adherence to the trial protocol. Data will be checked for missing or unusual values (range checks) and checked for consistency within participants over time. Discrepancies that have been raised will be queried.

The Trial Management Group will review rates of recruitment, missing outcome data, SAEs, study withdrawals and losses to follow-up across sites, and remedial action taken as necessary.

Completed paper Case Report Forms (CRFs) should be sent to the MC CTU promptly. The CTU will conduct data entry checks and use automated validation checks at data entry. A site visit by the Sponsor will be conducted if inconsistencies, unresolved queries, missing data are noted at a given site.

Monthly recruitment reports will be provided by the Sponsor, monitoring reasons cited for consent refusal and querying reasons for slow recruitment. The TMG is charged with providing solutions to problems where possible.

14.6 Confidentiality

Individual participant medical information obtained as a result of this study is considered confidential and disclosure to third parties is prohibited. CRFs will be labelled with a unique screening number or randomisation number.

14.7 Records Retention

The PI at each investigational site must make arrangements to store the essential trial documents, including the Investigator Site File, until the Sponsor informs the investigator that the documents are no longer to be retained, or for a maximum of 5 years, whichever is soonest. The five years is to be calculated from the date of last patient last point of trial contact.

In addition, the PI is responsible for archiving of all relevant source documents so that the trial data can be compared against source data after completion of the trial (e.g. in case of inspection from authorities).

The PI is required to ensure the continued storage of the documents, even if they leave the clinic/practice or retire before the end of the required storage period. Delegation should be documented in writing.

The MC CTU undertakes to store originally completed CRFs. All other essential documents will be stored by the CI/PI in the TMF/ISF.

15 Indemnity

The Sponsor, Alder Hey Children's NHS Foundation Trust, has insurance coverage for liabilities relating to harm caused by negligence in the design or management of the trial. The Sponsor does not provide cover for liabilities relating to non-negligent harm. Clinical negligence is covered by the standard NHS Indemnity provisions.

16 Financial Arrangements

This study is funded by the Research for Patient Benefit scheme of the National Institute for Health Research. Contractual agreements that incorporate financial arrangements will be in place between: the Sponsor and the University of Liverpool; the Sponsor and collaborating sites.

17 Trial Committees

17.1 Trial Management Group (TMG)

Membership of the TMG is listed in Appendix 2 of this protocol. The TMG will be responsible for the day-to-day running and management of the trial and will meet (in person or via teleconference/ videoconference) initially every month during trial setup and subsequently every 3 months once recruitment is underway.

17.2 Trial Steering Group (TSC)

Membership of the Trial Steering Committee is listed in Appendix 3 of this protocol. The role of the TSC is to provide overall supervision for the trial and provide advice through its independent Chairman. The ultimate decision concerning recommendations to the sponsor and funder about the continuation of the trial lies with the TSC.

18 Publication

The results from different centres will be collated and analysed together. Individual clinicians must undertake not to submit any part of their individual data for publication without the prior consent of the Trial Management Group.

The Uniform Requirements for Manuscripts Submitted to Biomedical Journals (http://www.icmje.org/) and the CONSORT guidelines will be respected. The ISRCTN allocated to this trial should be attached to any publications resulting from this trial.

BMJ guidance on authorship and contributorship (see http://bmj.com/advice/3.html) will be used to acknowledge the level and nature of contribution of key individuals in publications arising from the trial. The publication strategy shall lie under the jurisdiction of the Trial Steering Committee. Publication of results may be left until data from the planned definitive study has been completed.

19 Appendices

19.1 Appendix 1: Glossary

**A&E**	Accident and Emergency Unit
AE	Adverse Event
AR	Adverse Reaction
BTS/SIGN guidelines	British Thoracic Society guidelines on the management of asthma
CI	Chief Investigator
CONSORT	CONsolidated Standards of Reporting Trials
CRF	Case Report Form
CSP	NIHR Co-ordinated streamlined system for obtaining NHS permission
CTU	Clinical Trials Unit
Degree C	Degree Celsius
Eudract	European Union Drug Regulating Authorities Clinical Trials
GM	gram
GCP	Good Clinical Practice
HDU	High Dependency Unit
ICH	International Committee on Harmonisation (in GCP)
ISF	Investigator Site
ISRCTN	International Standard Randomised Controlled Trial Number
kg	kilogram
L/min	Litres per minute
LREC	Local Research Ethics Committee
MAGNETIC	Magnesium Sulphate Trial in Children
MC CTU	Medicines for Children Clinical Trials Unit
MREC	Multi-centre Research Ethics Committee
NHS	National Health Service
NIHR CRN	National Institute for Health Research Clinical Research Network
NIHR HTA	National Institute for Health Technology Assessment
NIHR RfPB	National Institute for Health Research Research for Patient Benefit funding stream
PI	Principal Investigator
PICU	Paediatric Intensive Care Unit
RCT	Randomised Controlled Trial
REC	Research Ethics Committee

TMG	Trial Management Group
TSG	Trial Steering Group
Yung's ASS	Yung's Asthma Severity Score

19.2 Appendix 2: Trial Management Group

Professor Paul McNamara, CI
Professor Matthew Peak, Director of Research AHFT and co-investigator
Dr Steven Lane, Statistician, MC CTU
Mrs Vanessa Compton, Clinical Specialist Physiotherapist, co-investigator
Mrs Christine Doyle, Consultant Nurse (Respiratory and Allergy) co-investigator
Dr Julie Grice, Consultant in paediatric emergency medicine – co-investigator
Mrs Tracy Moitt – Supervising Trials Manager, MC CTU
Mrs Janet Clark, lead research nurse AHFT
Mrs Samantha Jackson, lay research person (parent of children with asthma)
Miss Lucy Cooper, Research Governance and quality Assurance Lead

19.3 Appendix 3: Trial Steering Group

Please see appendix 3
