# Supplementary material for: Humidified and standard oxygen therapy in acute severe asthma in children (HUMOX): A pilot randomised controlled trial
Source: PLoS One. 2022 Feb 3;17(2):e0263044. doi: 10.1371/journal.pone.0263044 (PMC8812987; doi:10.1371/journal.pone.0263044)
Supplement: Attachment — Submitted filename: Response to Reviewers.docx [file pone.0263044.s011.docx]

**Responses to Reviewer’s Comments**

**Academic Editor Comments**

- **The use of randomisation envelope and block randomisation.**

**HUMOX RESPONSE:**

The use of stratified block randomisation is one that is both commonly used by trialists in the UK and also recommended by ICH [1] and as the reviewer points out, the use of block randomisation can lead to prediction. However, importantly, Clark et al highlight in the British Medical Journal that using variable block sizes can minimise the problem of selection and prediction bias [2].

We agree with the reviewer that the mis-use of randomisation envelopes in trials can lead to biases. However, Clark et al describe this process as being robust if ‘sequentially numbered opaque envelopes are truly opaque, sequentially numbered and opened in the correct order’, and such trials are described as having a low risk of bias in the Cochrane Handbook [2]. This was the process used in the HUMOX trial and there were regular checks by the Research Nurse and the statistical team to ensure that all envelopes were opened in the correct order and all envelopes were returned at the end of the trial and showed no signs of being tampered with. We are fully aware that this method does not ensure that the seals have not been broken previously but such suggestions would indicate professional mis-conduct, the occurrence of which is rare in clinical trials [3] and we had no indication to think such occurrences had taken place in the HUMOX trial.

We understand the concerns raised by the reviewer but believe that this manuscript is not the place to describe in detail the methodology of randomisation and allocation concealment. However, we do agree with CONSORT that there should be enough information for the reader to make an informed decision regarding the methods used in a trial and feel that as authors of this manuscript we have fulfilled this obligation.

- **What [was] the plan for dealing with missing data?**
- **How [were] the data analyzed?**

**HUMOX RESPONSE:**

We have chosen to answer both these points in one response. When planning the analysis for the HUMOX trial we carefully consider the recommendations by Lancaster et al, which clearly state that the ‘The analysis of any type of pilot study should be mainly descriptive or should focus on confidence intervals’ [5]. These recommendations were followed in the HUMOX trial. Consequently, we did not conduct any hypothesis tests and only reported summary statistics and 95% confidence intervals; it has previously been reported that many pilot studies place an unnecessary emphasis on hypothesis testing [6]. This is clearly defined in the methods section of the manuscript and we would be happy to publish the full statistical analysis plan alongside the manuscript.

Missing data were presented as a proportion of data that had been reported and in line with the recommendations above; there was no statistical testing and no imputation was carried out as it was felt that it was not appropriate given the objectives of this pilot study. This was again described in the methods section of the manuscript.

- **Why are there no p-values in Table 1?**

**HUMOX RESPONSE:**

One of the many recommendations of the CONSORT statement is that a table should be presented showing baseline demographic and clinical characteristics for each group. Importantly, according to this CONSORT recommendation [4], “significance testing of baseline differences in randomized controlled trials (RCTs) should not be performed, because it is superfluous and can mislead investigators and their readers”. Again, we have followed the internationally recognised reporting standards of clinical trials and not conducted hypothesis tests on baseline data. This again was discussed in the methods section of the manuscript.

**References**

[1] McPherson, G.C., Campbell, M.K. & Elbourne, D.R. Use of randomisation in clinical trials: a survey of UK practice. *Trials* **13,** 198 (2012). <https://doi.org/10.1186/1745-6215-13-198>

[2] Clark L, Fairhurst C, Torgerson DJ. Allocation concealment in randomised controlled trials: are we getting better? BMJ. 2016 Nov 17;355:i5663. doi: 10.1136/bmj.i5663. PMID: 27856428.

[3] George SL. Research misconduct and data fraud in clinical trials: prevalence and causal factors. Int J Clin Oncol. 2016 Feb;21(1):15-21. doi: 10.1007/s10147-015-0887-3. Epub 2015 Aug 20. PMID: 26289019.

[4] Moher D, Hopewell S, Schulz KF, Montori V, Gøtzsche PC, Devereaux PJ, et al. CONSORT 2010 Explanation and Elaboration: Updated guidelines for reporting parallel group randomised trials. J Clin Epidemiol. 2010;2010(63):e1–37.

[5] Lancaster GA, Dodd S, Williamson PR. Design and analysis of pilot studies: recommendations for good practice. J Eval Clin Pract. 2004 May;10(2):307-12. doi: 10.1111/j..2002.384.doc.x. PMID: 15189396.

[6] Arain M, Campbell MJ, Cooper CL, Lancaster GA. What is a pilot or feasibility study? A review of current practice and editorial policy. BMC Med Res Methodol. 2010 Jul 16;10:67. doi: 10.1186/1471-2288-10-67. PMID: 20637084; PMCID: PMC2912920.

Additional Requirements

1. Please ensure that your manuscript meets PLOS ONE's style requirements, including those for file naming. The PLOS ONE style templates can be found at

<https://journals.plos.org/plosone/s/file?id=wjVg/PLOSOne_formatting_sample_main_body.pdf> and

<https://journals.plos.org/plosone/s/file?id=ba62/PLOSOne_formatting_sample_title_authors_affiliations.pdf>

**HUMOX RESPONSE:**  This has been addressed.

2. Please ensure that all figures and tables have been corrected referenced in-text.

**HUMOX RESPONSE:** The text of the manuscript has been checked to ensure that all figures and table are correctly referenced in the text.

3. In your Methods section, please provide additional information about the participant recruitment method and the demographic details of your participants. Please ensure you have provided sufficient details to replicate the analyses such as: a) a description of how participants were recruited, and b) descriptions of where participants were recruited and where the research took place.

**HUMOX RESPONSE: Further information regarding how and where participants were recruited have now been added to the manuscript.**

4. Please provide additional details regarding participant consent. In the ethics statement in the Methods and online submission information, please ensure that you have specified (1) whether consent was informed and (2) what type you obtained (for instance, written or verbal, and if verbal, how it was documented and witnessed). Since your study included minors, please also state whether you obtained consent from parents or guardians.

**HUMOX RESPONSE:** A revised section on consent has been added to the manuscript.

5. In your Data Availability statement, you have not specified where the minimal data set underlying the results described in your manuscript can be found. PLOS defines a study's minimal data set as the underlying data used to reach the conclusions drawn in the manuscript and any additional data required to replicate the reported study findings in their entirety. All PLOS journals require that the minimal data set be made fully available. For more information about our data policy, please see <http://journals.plos.org/plosone/s/data-availability>.

Upon re-submitting your revised manuscript, please upload your study’s minimal underlying data set as either Supporting Information files or to a stable, public repository and include the relevant URLs, DOIs, or accession numbers within your revised cover letter. For a list of acceptable repositories, please see <http://journals.plos.org/plosone/s/data-availability#loc-recommended-repositories>. Any potentially identifying patient information must be fully anonymized.

Important: If there are ethical or legal restrictions to sharing your data publicly, please explain these restrictions in detail. Please see our guidelines for more information on what we consider unacceptable restrictions to publicly sharing data: <http://journals.plos.org/plosone/s/data-availability#loc-unacceptable-data-access-restrictions>. Note that it is not acceptable for the authors to be the sole named individuals responsible for ensuring data access.

 We will update your Data Availability statement to reflect the information you provide in your cover letter.

**HUMOX RESPONSE:** The Data Availability section has been amended to make the point of contact the central Clinical Trials Unit account and not an individual’s email.

6. PLOS requires an ORCID iD for the corresponding author in Editorial Manager on papers submitted after December 6th, 2016. Please ensure that you have an ORCID iD and that it is validated in Editorial Manager. To do this, go to ‘Update my Information’ (in the upper left-hand corner of the main menu), and click on the Fetch/Validate link next to the ORCID field. This will take you to the ORCID site and allow you to create a new iD or authenticate a pre-existing iD in Editorial Manager. Please see the following video for instructions on linking an ORCID iD to your Editorial Manager account: <https://www.youtube.com/watch?v=_xcclfuvtxQ>

**HUMOX RESPONSE:** The ORCID ID has been added for the corresponding author.

7. Your ethics statement should only appear in the Methods section of your manuscript. If your ethics statement is written in any section besides the Methods, please delete it from any other section.

**HUMOX RESPONSE:** This has now been included in the methods section under ‘Approvals’.

8. Please ensure that you refer to Figures 1 and 2 in your text as, if accepted, production will need this reference to link the reader to the figure.

**HUMOX RESPONSE:** All tables and figures are now referred to in the text as are supplementary material.

9. We note you have included a table to which you do not refer in the text of your manuscript. Please ensure that you refer to Table 2 in your text; if accepted, production will need this reference to link the reader to the Table.

**HUMOX RESPONSE:** All tables and figures are now referred to in the text as are supplementary material.

10. Please include captions for your Supporting Information files at the end of your manuscript, and update any in-text citations to match accordingly. Please see our Supporting Information guidelines for more information: [http://journals.plos.org/plosone/s/supporting-information](about:blank).

**HUMOX RESPONSE:** The information in regards to supporting information has been included at the end of the manuscript.

**Reviewers' comments**

**Abstract
Page 3 Line 39-40, the sentence ‘ (shortest for standard O2, 37.9(29.1) hours) and 14.6 (14.2) hours (shortest for cold humidified O2, 13.1(14.9) hours) respectively’ to be revised and to state for all groups with their respective Median ±IQR.**

**HUMOX RESPONSE:**  The results for the three groups have been added as requested.

**Page 3 Line 41, for ‘inhaled treatment was 31.4 (22.2) hours, the group name to be stated. Likewise to include other groups readings**.

**RESPONSE:** The results for the three groups have been added as requested.

**Introduction
Page 5, Introduction was too short. More information to be provided.**

**HUMOX RESPONSE: Further information has been provided in the introduction**

**Methods
Recruitment
Page 6, more information to be provided on how the subjects were recruited (as illustrated in Figure 1) in the method section.**

**HUMOX RESPONSE:** Further information has been provided regarding how subjects were recruited in the manuscript.

**Randomization
Page 7 Line 95-96, more description to be provided for this sentence ‘Stratified block randomisation (age (2-5 years and 6-16 years) and centre, block sizes of 3 and 6)’**

**HUMOX RESPONSE:** Further information has now been provided regarding randomisation.

**Outcome measures
Page 8 Line 129, full name for abbreviation ASS (Asthma Severity Score) to be stated.**

**HUMOX RESPONSE:** This has now been corrected.

**Data analysis
Page 9 Line 141, statistical software including version and publisher name which was used to perform the descriptive statistics to be stated.**

**HUMOX RESPONSE:** This has now been added to the manuscript.

**More information on missing data to be provided in terms of percentage, pattern etc.**

**HUMOX RESPONSE:**  Missing data information have been provided in the main table in the manuscript and also in the tables contained in the supplementary table. The discussion section highlights the fact that an outcome (PRAM) was dropped due the high level of missing data.


**Results
Page 10 Line 157, incomplete sentence.**

**HUMOX RESPONSE:** This has now been corrected.

**Page 10 Line 158-162 and 164-168, content were similar and repeated**.

**HUMOX RESPONSE:** Apologies we are not sure which text the reviewer thinks is being repeated.

**Page 11-12, Line 180-181, sentence missing**.

**HUMOX RESPONSE:** The section in regards to PRAM has been re-worded.

**Page 13 Line 194-199, separate groups findings to be provided.**

**HUMOX RESPONSE:**  Data were measured across several time points during the period of the trial and detail on these time points is provided in the supplementary tables, we did not feel it appropriate to pick an arbitrary time point for the main paper and have therefore made a general statement about the results.

**Page 10 & 14 Line 158, Line 164, Line 216, there were many ‘Error! Reference source not found:’**

**HUMOX RESPONSE:** We were unable to see these error messages in the document that we submitted.

**There were many missing data/not assessed and this needs to be discussed.**

**HUMOX RESPONSE**: We have added a further paragraph into the discussion relating to missing data and the planning of a future trial.

**Table 1, the title can be expanded. At least 1 decimal point for the percentage figures. In the table footnote, it was stated ‘***=3 values missing’ but was not found in the table. Range to be replaced with IQR.**

**HUMOX RESPONSE:**  All these changes have been made to Table 1.

**Table 2, the readings at baseline, 2-hour, 4-hour, 6-hour, 8-hour and 12-hour to be provided before deriving the mean difference. Intent to treat and n to be stated.**

**HUMOX RESPONSE:** Table 2 has now been updated to include the requested information.

**Figure 1, the assessment period to be incorporated in and intention to treat to be stated.**

**HUMOX RESPONSE:** The time period that patients were screened in has been added.

**Figure 2, n to be stated. There were two titles. Title in the graph to be removed.**

**HUMOX RESPONSE:** Figure 2 has now been updated as requested.
