# Supplementary material for: Humidified and standard oxygen therapy in acute severe asthma in children (HUMOX): A pilot randomised controlled trial
Source: PLoS One. 2022 Feb 3;17(2):e0263044. doi: 10.1371/journal.pone.0263044 (PMC8812987; doi:10.1371/journal.pone.0263044)
Supplement: Attachment — Submitted filename: Response to Editor Comments.docx [file pone.0263044.s012.docx]

| Editor Comments |  |
| --- | --- |
| 1. A statement in the abstract along the lines of: Because of the small sample size of this pilot, conclusions cannot be extrapolated beyond the study sample.” So that from the beginning readers are not looking for inferential conclusions. | We have added a statement in the ‘Conclusions’ of the abstract highlighting this point. |
| 2. A paragraph in the methods section explaining to readers the purpose of a pilot study such as yours. In addition to your reference 9 (Lancaster et al, 2004), consider using the following: <https://www.nccih.nih.gov/grants/pilot-studies-common-uses-and-misuses> <https://www.ncbi.nlm.nih.gov/pmc/articles/PMC3081994/> <https://www.ncbi.nlm.nih.gov/pmc/articles/PMC4917389/> | We have included a paragraph to highlight the purpose of a pilot study and referenced the paper by Leon. |
| 3. A paragraph in the discussion about what you will do to improve the randomization in a future confirmatory trial. For instance, increase the number of random block sizes to 3, 6, 9, 12, and 15, or use a different randomization method altogether, and implement a computerized system (as opposed to the envelopes). | We have included a paragraph in the discussion that highlights that consideration needs to given to method of allocation concealment in trials conducted in an emergency setting. |
| 4. Minor fix: typo in abstract results: “[…]time (hours) taken to step down nebulised to inhaled treatment was 5.6 (14.3),” should be 35.6 (14.3). | We have corrected this. |
| Reviewer 2 comments |  |
| 1. The Procedures section specifies the duration of assessment to be "as long as they required O2 (and until discharge)". Do the authors mean that all the patients were followed until their discharge? Alternatively, this sentence could mean the patients were followed until they were either off O2 or had been discharged. This sentence should be rewritten to be more clear. | We have re-wrote this sentence to ensure that it is clearer. |
| 2. The Procedures mention that the parents or guardians provided recommendations regarding potential meaningful patient outcomes and post-A&E Department therapy. The details regarding these findings are provided in the Supplemental Material. However, no reference to this is made within the Procedures section. The authors should state that these findings are provided in the Supplemental Material appendix to make it easier for the interested reader to locate them. | The results section refer to where these findings are in the Supplementary material. |
| 3. The authors state that the outcomes were not designated as primary or secondary, but do not provide a rationale for doing that. The authors should provide a rationale as to why they chose to not designate outcomes as primary or secondary. | We have highlighted that due to the fact that this study was a pilot study and there was no hypothesis testing then there were no primary or secondary outcomes. |
| 4. The authors should provide more details regarding how they arrived at 30 per group as their sample size. Did they conduct a power analysis? Was this based on the average number of children seen in the A&E Department for acute asthma exacerbations? | We have stated in the manuscript that the size of the sample size was based on pragmatism and referenced appropriately. There was no power analysis completed as one of the objectives was to collect information to enable this for a future trial, which we write about in the discussion. |
| 5. The Discussion outlines some of the barriers experienced by the authors in obtaining their data. While they report some of the approaches that were not successful, it would be helpful if they would include potential ways that they might be successful in collecting a more complete data set in a subsequent study. This is particularly important since the difficulties with missing data precluded the use of a clinically relevant assessment, the PRAM score. | We have included a paragraph in the discussion that highlight strategies that may improve data collection during the course of a larger RCT. |
| Reviewer 3 |  |
| : In the table 1, when you compare the three groups with parametric method do you use to write they are similar? Can you explain why did not carried out hypothesis testing? | One of the many recommendations of the CONSORT statement is that a table should be presented showing baseline demographic and clinical characteristics for each group. Importantly, according to this CONSORT recommendation [1], “significance testing of baseline differences in randomized controlled trials (RCTs) should not be performed, because it is superfluous and can mislead investigators and their readers”. We have followed the internationally recognised reporting standards of clinical trials and not conducted hypothesis tests on baseline data. This was discussed in the methods section of the manuscript.  [1] Moher D, Hopewell S, Schulz KF, Montori V, Gøtzsche PC, Devereaux PJ, et al. CONSORT 2010 Explanation and Elaboration: Updated guidelines for reporting parallel group randomised trials. J Clin Epidemiol. 2010;2010(63):e1–37. |
